# In Vivo Administration of Scallop GnRH-Like Peptide Influences on Gonad Development in the Yesso Scallop, *Patinopecten yessoensis*

**DOI:** 10.1371/journal.pone.0129571

**Published:** 2015-06-01

**Authors:** Kazue Nagasawa, Hitoshi Oouchi, Naoki Itoh, Keisuke G. Takahashi, Makoto Osada

**Affiliations:** 1 Laboratory of Aquacultural Biology, Graduate School of Agricultural Science, Tohoku University, 1–1 Amamiya-machi, Tsutsumidori, Aoba-ku, Sendai, Miyagi, Japan; 2 Graduate School of Agricultural and Life Sciences, The University of Tokyo, 1-1-1, Yayoi, Bunkyo-ku, Tokyo, Japan; Florida International University, UNITED STATES

## Abstract

Existing research on the role of gonadotropin-releasing hormone (GnRH) in bivalve reproduction is inadequate, even though a few bivalve GnRH orthologs have been cloned. The objective of this paper was to elucidate the in vivo effect of GnRH administration in Yesso scallop reproduction. We performed in vivo administration of scallop GnRH (py-GnRH) synthetic peptide into the developing gonad, and analyzed its effect on gonad development for 6 weeks during the reproductive season. The resulting sex ratio in the GnRH administered (GnRH(+)) group might be male biased, whereas the control (GnRH(-)) group had an equal sex ratio throughout the experiment. The gonad index (GI) of males in the GnRH(+) group increased from week 2 to 24.8% at week 6. By contrast the GI of the GnRH(-) group peaked in week 4 at 16.6%. No significant difference was seen in female GI between the GnRH(+) and GnRH(-) groups at any sampling point. Oocyte diameter in the GnRH(+) group remained constant (about 42–45 μm) throughout the experiment, while in the GnRH(-) group it increased from 45 to 68 μm i.e. normal oocyte growth. The number of spermatogonia in the germinal acini of males in the GnRH(+) group increased from week 4 to 6. Hermaphrodites appeared in the GnRH(+) group in weeks 2 and 4. Their gonads contained many apoptotic cells including oocytes. In conclusion, this study suggests that py-GnRH administration could have a potential to accelerate spermatogenesis and cause an inhibitory effect on oocyte growth in scallops.

## Introduction

A clear neuroendocrinological regulation of vertebrate gonadotropin releasing hormone (GnRH) controlling gonadal maturation can infer possible presence of ancestral molecule for regulating reproduction in invertebrates even though neither their GTH-like hormone nor secreting organ have been fully identified yet. For a great interest in invertebrate (inv) GnRH-like peptides, a number of GnRHs and their receptors (GnRHRs) have been identified in both Deuterostomia and Protostomia [1]. By a genome-wide in-silico mining and comparative analysis, Roch et al. [2, 3] recently proposed a hypothesis that a common ancestor of the GnRH super family (i.e., vertebrate GnRHs, invGnRHs, corazonins (Crzs), adipokinetic hormones (AKHs), AKH/Crz related peptide (ACP)) had presented in the ancestral species. Since their analysis revealed that only amphioxus genome possesses GnRHRs and invGnRHRs that both can recognize and act with their individual ligands, therefore they suggested the gene duplication occurred in the ancestral bilaterian resulting two receptors (i.e., GnRHR/AKH receptor and invGnRHR/Crz receptor) with their own ligands [2]. Consequently, throughout the evolution process, vertebrates lost both invGnRH and invGnRHR and take over the other one set, whereas invertebrate Protostomia may only possesses the signaling pathway mediated with invGnRH and invGnRHR. Nevertheless, a longstanding question is still at issue whether Protostomian GnRHs (invGnRHs) are true orthologs to Deuterostomian GnRHs due to limited number of physiological analyses in vivo [1].

In invertebrate Protostomia, several physiological analyses for GnRHs have been mainly studied in mollusk and proposed that GnRH may have a function to activate the reproduction [4–7], but the point in controversy is that most physiological studies validated the influence with vertebrate-type GnRH peptides, which would need to interpret in consideration of ligand functionality to invGnRHR. However, a few of the precise investigations with endogenous peptide sequences speculated the possible function of invGnRHs in mollusks, proposing its diverse role with severalty among taxa. In vivo administration studies in Gastropoda using *Aplysia californica* revealed that Aplysia GnRH (ap-GnRH) failed to affect any reproductive parameters (e.g., reproductive organ mass, oocyte growth, penile eversion, egg-laying activity) and the release of egg-laying hormone (ELH) which can stimulate the ovulation and egg-laying behavior [8, 9]. Instead, its non-reproductive role has been proposed that ap-GnRH may modulate muscular contraction for controlling parapodia, foot, and head movement [10]. Another functional evidence of mollusk GnRH could be found in Cephalopoda: Bioassay with *Octopus vulgaris* GnRH (oct-GnRH) for monitoring cardiac activity and spontaneous contraction of the oviducts identified its modulatory effects on the contractions of the heart [11] and oviducts [12] suggesting its multifunctional modulatory effects. Especially for its reproductive role, an organ culture study revealed that oct-GnRH directly stimulates steroid production of the immunoassayable progesterone (P), testosterone (T), and 17β-estradiol (E_2_) in both octopus testis and ovary [13], resulting in an increase of ovarian weight in the annual reproductive period [14, 15]. These observations from the cephalopod are only functional evidences that suggest the reproductive role of invGnRH which can promote gonad development by stimulating the release of sex steroids as vertebrates do.

In bivalves, there has been no functional evidence to validate the invGnRH function in vivo. However, attempts with molecular-based identification for invGnRH have been strenuously carried out in a few bivalve species. Intriguingly, a recent study using mass spectrometry determined that two types of GnRH-like peptides exist in Pacific oyster *Crassostrea gigas* [16]: pQNYHFNSNGWQP-NH_2_ (cg-GnRH-a), an amidated undecapeptide, and pQNYHFNSNGWQPG (cg-GnRH-G), a non-amidated dodecapeptide. In Yesso scallop *Patinopecten yessoensis*, Treen et al. [17] cloned a scallop GnRH (py-GnRH) cDNA sequences and proposed the possible presence of two forms of mature py-GnRH peptide sequences (pQNFHYSNGWQP-NH_2_ (py-GnRH-P-NH_2_), an amidated undecapeptide and pQNFHYSNGWQPG-NH_2_ (py-GnRH-G-NH_2_), an amidated dodecapeptide that may be generated by cutting a signal peptide and GnRH-associated peptide based on a conceptual cleavage at typical processing. Though both peptide forms possess an activity to promote spermatogonial proliferation in vitro, the shorter form (py-GnRH-P-NH_2_) is thought to be more logically a natural product from the biochemical amidation process [17, 18]. In addition, organ culture studies with scallop gonads revealed that both m-GnRH and the tissue extract of cerebral and pedal ganglion (CPG) could stimulate proliferation of spermatogonia but not oogonia [5], suggesting that py-GnRH may have a potential to promote male gonad development [19].

At the present stage, limited knowledge of functional evidences about invGnRH still tells us that they do not have uniformity of neither reproductive role nor other physiological role. To assess the in vivo invGnRH function in bivalves, we administered the synthetic py-GnRH peptide by a slow-release system into the gonad. Scallops are dioecious bivalves and have gonads that are clearly separate from the other organs. Hence their sex can be distinguished during the reproductive season by the color of the gonads without dissection. Therefore *P*. *yessoensis* was chosen as a suitable representative species to investigate the influence of invGnRH administration in terms of ease of injecting substrate, subsequent sexing, and weighing the gonads. We also assessed the long-term effect of py-GnRH administration on gonad development and sex differentiation during a reproductive season by using histological observation with apoptotic analysis.

## Materials and Methods

### Preparation of the emulsion of synthetic py-GnRH peptide

Synthetic py-GnRH peptide (pQNFHYSNGWQP-NH_2_) with a molecular weight of 1359.38 and 36% hydrophobicity was generated by Sigma-Aldrich (St. Louis, MO, USA) and dissolved with distilled water at 10^–3^ molar as a stock solution. To prepare an emulsion, the stock solution was diluted with an equal volume of 100% EtOH, and then added to 10 times the amount (v/v) of molten cacao butter at around 45°C, resulting in a final concentration of synthetic peptide of 70 μg/ml (5•10^–5^ molar). The control emulsion was prepared with the same volume of 50% EtOH without synthetic peptide.

### Administration of GnRH-related peptide into scallop gonad

**Fig 1:**
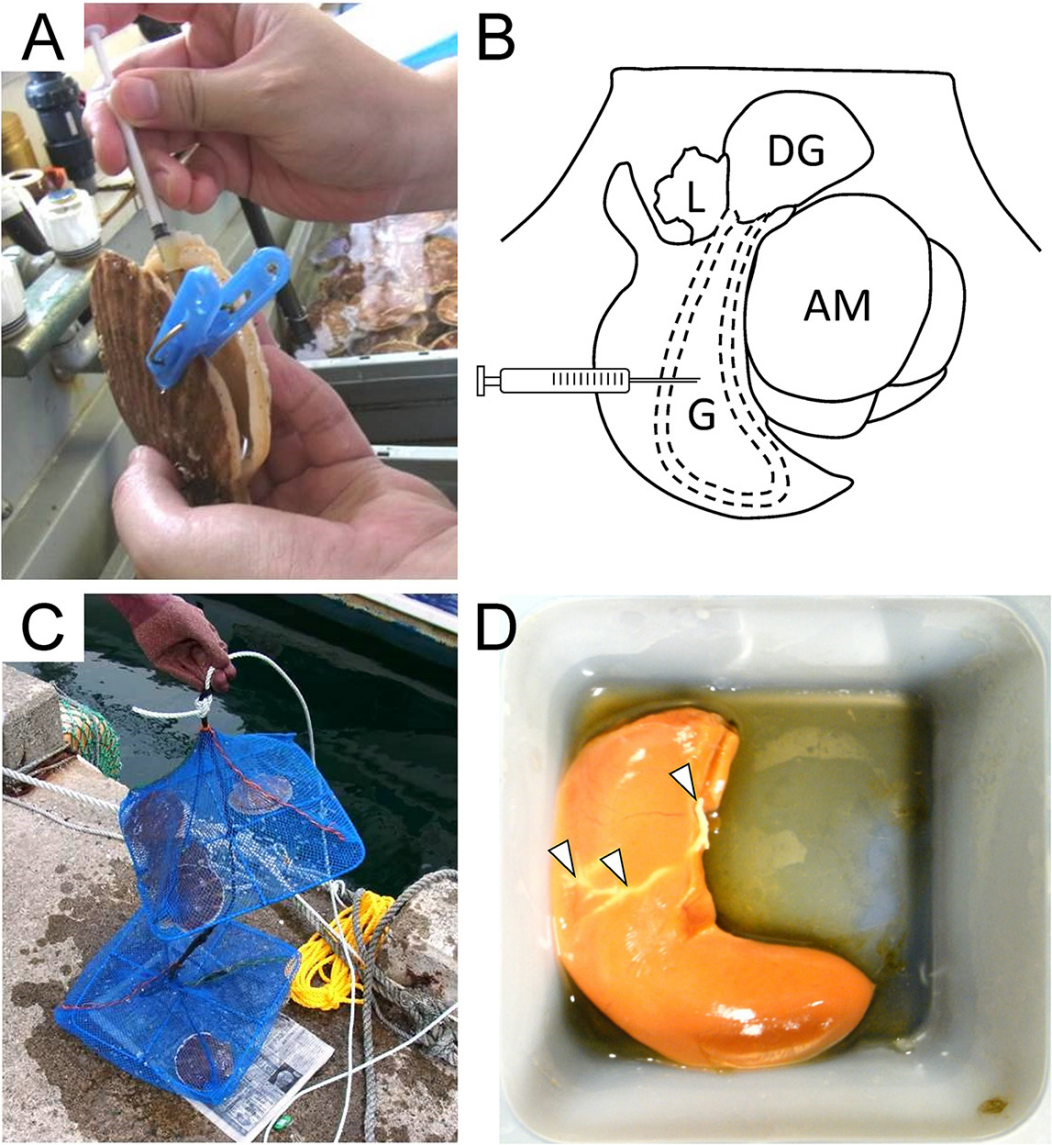
Administration with emulsion containing py-GnRH peptide into scallop gonad. A) Shells clamped with clothespin to expose the gonad for injection with emulsion. B) Schematic diagram indicating the position of injection. Gonad intestinal loop is indicated with dashed line in the gonad. AM, adductor muscle; DG, digestive gland; G, gonad; L, lips. C) The treated scallops were placed in net cages for rearing. D) Excised ovary with solidified emulsion taken from the scallop reared for 6 weeks. Arrowheads indicate the solidified emulsion remaining in the gonad.

Two-year old cultured Yesso scallops *Patinopecten yessoensis*, were purchased from the commercial supplier in Onagawa Bay, Miyagi, Japan. The gonad of each scallop received an implantation of py-GnRH peptide. Shells were partially opened by an opener (gag) and clamped with a clothespin to keep the shells open to expose the gonad (Fig 1A). Then, 200 μl of the emulsion containing 14 μg of py-GnRH was injected by a 1 ml syringe with a 15 gauge needle into the gonad (Fig 1B), while the control group received an injection of the same volume of emulsion without the synthetic py-GnRH peptide. The injected emulsion immediately solidified in the scallop gonad when the emulsion was cooled below 10°C. Then, the treated scallops were kept in net cages and placed in hanging type facilities for culture with other commercial scallops in Onagawa Bay for 6 weeks (Fig 1C). The soft body tissue and gonad were subsequently excised, weighed for calculation of gonad index (GI, 100•gonad weight/soft body weight (%)), and sampled for fixation for sexing and further histological analysis 0, 2, 4, and 6 weeks after implantation.

### Histological analysis for measurement of oocyte diameter and count of spermatogonia

The methodology for measurement of oocyte diameter and count of spermatogonia were previously established as detailed elsewhere (Osada et al., 2007). In brief, gonads were excised from scallops and fixed with Davidson’s fixative at 4°C overnight. Fixed tissues were dehydrated, embedded in paraffin wax, and sectioned at 5 μm thickness. The paraffin sections were then mounted on glass slides (Matsunami Glass Ind., Ltd., Osaka, Japan), deparaffinized, and dehydrated by immersion in a Lemosol (Wako Pure Chemical Ind., Ltd., Osaka, Japan)-ethanol series. The sections were stained with hematoxylin-eosin (H.E.). For the measurement of oocyte diameter, 10 oocytes for 10 different area (i.e., 100 oocytes) for each specimen were randomly chosen and measured in the long axis direction by using cellSens Standard 1.7 software (Olympus, Tokyo, Japan) after capturing images by microscopy (BX-53; Olympus). Spermatogonia which exhibited a relatively large cell diameter were counted manually in 10 randomly chosen germinal acini for each specimen. Regarding the gonad of hermaphrodite scallops, both germinal acini containing oocytes and spermatogonia found in several different areas were analyzed individually as mentioned above.

### TUNEL assay

Apoptotic cells in gonads were assessed by the terminal deoxynucleotidyl transferase-mediated d-UTP nick end labeling (TUNEL) assay by using ApopTag Plus Peroxidase *In Situ* Apoptosis Kit (Millipore, MA, USA) following the manufacturer’s instructions. In brief, dehydrated sections at 5 μm thickness were permeabilized with ProK (20μg/ml) for 15 min at room temperature (RT), quenched with 3% hydrogen peroxide/PBS for 5 min at RT and equilibrated with equilibration buffer. Fragmented DNA on the section was labeled with digoxigenin (DIG)-labeled dNTPs by terminal deoxynucleotidyl transferase (TdT) activity for 1 hour at 37°C and then the hapten was immunodetected by horse radish peroxidase (HRP)-labeled anti DIG antibody for 30 min at RT and visualized with 3,3′-diaminobenzidine (DAB) substrate.

### Statistical analysis

To assess the effects of py-GnRH administration with the restricted specimen number, careful statistical analyses were conducted. For the changes in GI, oocyte diameter, and number of spermatogonia, all variables were tested for distribution normality using the Kolmogorov-Smirnov and the Shapiro-Wilks tests. Since some samples were not normally distributed, non-parametric statistics were conducted for all data. For the comparison of means within the same treatment group, one-way ANOVA (Kruskal-Wallis test) was used. If the one-way ANOVA was significant, the Dunn’s multiple comparison was used as a post hoc test. For the comparison of means between GnRH(+) and GnRH(-) groups at a particular sampling point, two-way ANOVA was used as long as each group has more than four individuals. If the two-way ANOVA was significant, the Bonferroni posttests were performed as a post hoc test. The sex ratio of scallops in the GnRH(+) and GnRH(-) groups was statistically analyzed by the chi-square test (S1 Table). For all statistical tests, significance levels were set at p < 0.05. As for the one-way ANOVA in GIs of hermaphrodites, a statistical test was not possible owing to the low group numbers (less than two groups).

## Results

### Biological effect of emulsion administration into scallop gonads

In total, 115 scallops received an administration of emulsion in January 2012 and were then placed in cages suspended with ropes at approximately 5 to 10 meters depth in Onagawa Bay, Miyagi prefecture, Japan for 6 weeks. After injection, molten emulsion was immediately solidified and anchored inside of gonads until the end of the experiment. Anatomical observation revealed that the site of necrosis was rarely found in gonadal tissue in contact with solidified emulsion, but all gonads with the implant had healthy growth throughout the experiment (Fig 1D). During six week, mortality (approximately 10% of total number) was found in week 2 to 4 for both treatment groups (S1 Table). Eventually, a total of 73 scallops (63.4% of initial number) that had received an administration survived and were sampled for weighing and further histological analysis. Our time-resolved fluoresce immunoassay (TR-FIA, S1 Fig) was able to measure py-GnRH in the range from 1.36 μg/ml to 13.6 pg/ml. Py-GnRH was measurable from hemocyte extracts of the py-GnRH(+) group in week 2 whereas py-GnRH was not detectable from plasma in any individuals throughout the experiment (S1 Fig).

### Shift in sex ratio throughout the experiment

**Fig 2:**
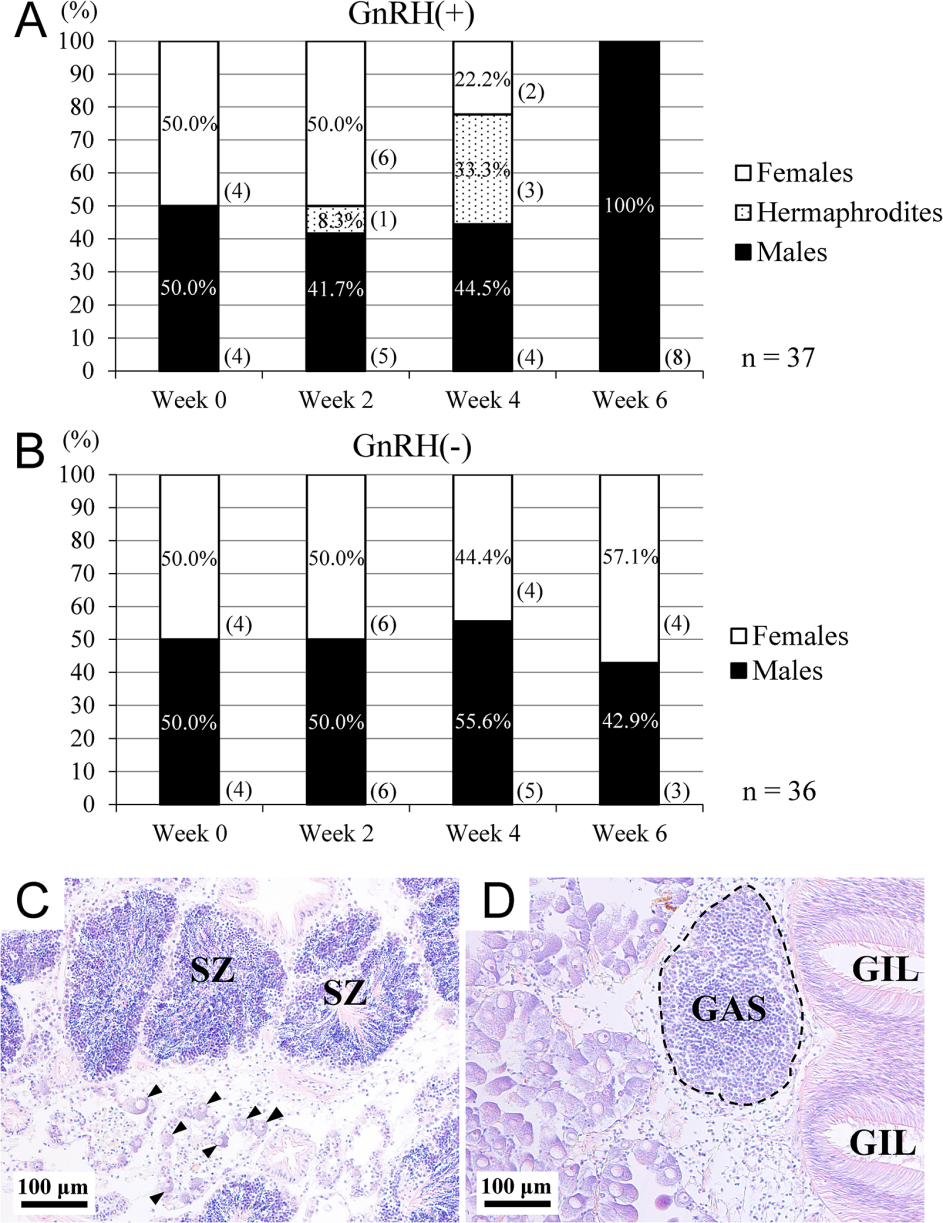
Sex ratio of scallops after py-GnRH administration during 6 weeks. Scallops received an implantation of emulsion with (A, GnRH(+), n = 37) and without synthetic py-GnRH peptide (B, GnRH(-), n = 36), were sampled, and sex determined at week 0, 2, 4 and 6. The numbers in parenthesis beside the columns indicate individual number for each sex. A) In the GnRH(+) group, hermaphrodites were found in week 2 and 4. B) In the GnRH(-) group, an almost equal number of each sex was found at all sample times. C, D) Histological observation with hematoxylin and eosin (H.E.) staining of the gonad excised from a hermaphrodite in the GnRH (+) group. C) Arrowheads indicate oocytes. SZ; spermatozoa. D) The germinal acinus containing spermatogenic cells (GAS) is encircled with a dashed line. GIL; gonad intestinal loop.

To determine the *in vivo* effect of py-GnRH administration on sexuality, sex was confirmed by both visual judgment and histological observation of germ cell type in gonads. In general, the sex ratio of intact samples was not biased prior to implantation. However, a chi-square test revealed that there must be a relationship between administration of py-GnRH and the sex ratio throughout the experiment (p = 0.027 in S1 Table) and the assumption of independence in the GnRH(+) group is invalid, whereas no relationship was observed in the GnRH(-) group (p = 0.968 in S1 Table) and the assumption of independence could not be rejected. Specifically, at the initial sampling point at week 0 (Fig 2A and 2B), an equal number of males and females were found in both groups receiving an injection of py-GnRH peptide (GnRH(+)) or the blank (control, GnRH(-)). In the GnRH(+) group at week 2, the sex ratio of males and females was almost even (Fig 2A). However, the histological observation of gonads stained by hematoxylin-eosin (H.E.) revealed that there was only one scallop that was a hermaphrodite possessing both sperm and oocytes at week 2 in the GnRH(+) group (Fig 2A). In addition to the GnRH(+) group, the ratio of hermaphrodites had increased from 8.3% in week 2 to 33.3% in week 4. In hermaphrodite gonads, the typical histological morphology of oocytes and spermatogenic cells was observed (Fig 2C and 2D). Meanwhile, the ratio of females (22.2%) was lower than males (44.5%) at week 4. In the GnRH(+) group at week 6, the sampled scallops were all males (Fig 2A). No difference in the sex ratio was found in the control group at any of the sampling times (Fig 2B).

### Gonad index (GI) changes

**Fig 3:**
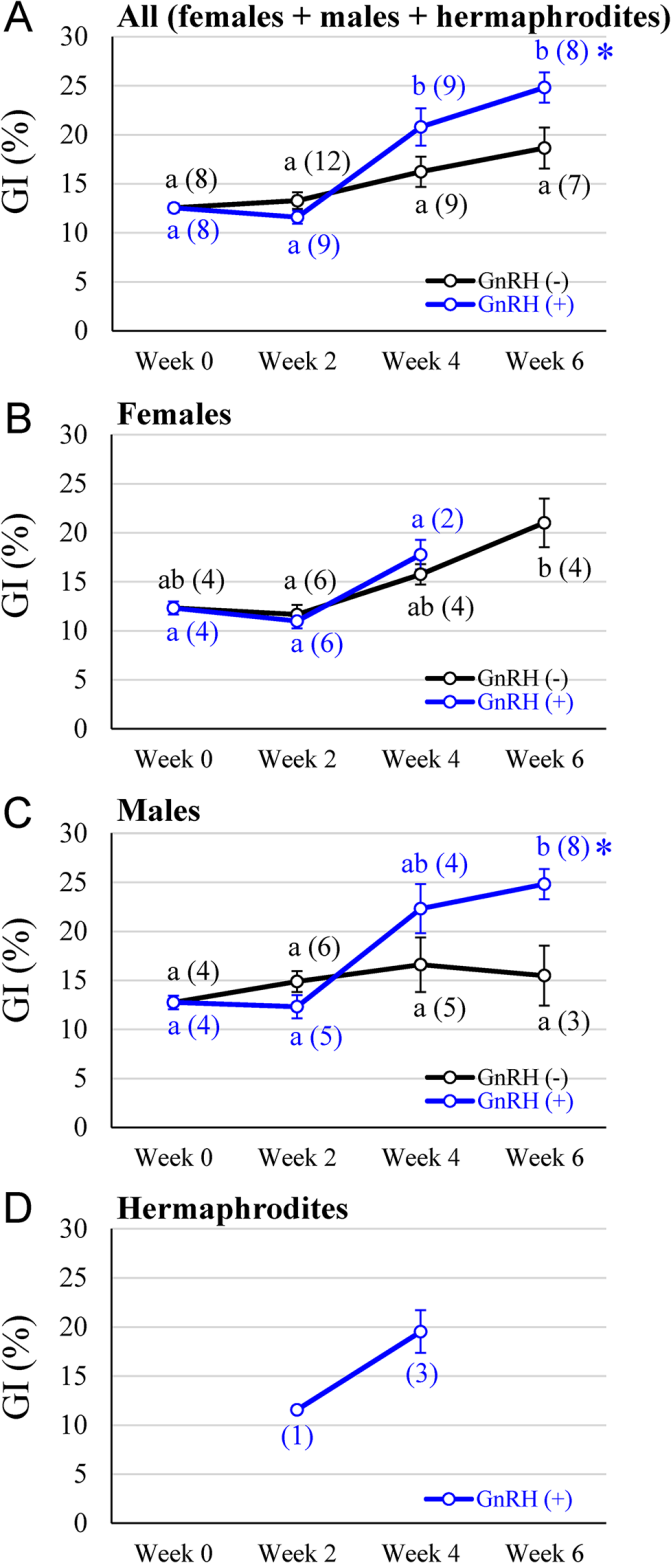
Changes in gonad index (GI) in the scallops after py-GnRH administration during 6 weeks. The GIs for all specimens (A), females (B), males (C) and hermaphrodites (D) were assessed separately. Black and blue lines for the GnRH(-) and GnRH(+) groups, respectively. All data are presented as a mean ± standard error of the mean. Different superscript letters indicate significant differences within the same treatment group. The parenthetical references indicate specimen number for each group assessed. Asterisks (*) indicate significant differences in the GnRH(+) group compared to GnRH(-) group at a particular sampling point at p < 0.05.

To assess the effect of py-GnRH administration on gonad development, gonad index (GI) was calculated for all specimens sampled. The initial GI values did not differ between GnRH(+) and GnRH(-) groups in week 0 (Fig 3A–3C). In Fig 3A, the GI of the GnRH(-) group slowly increased until the end of experiment without any significant differences among all sampling times, whereas, the GI of the GnRH(+) group increased significantly from week 2 to 4, and kept on increasing until week 6. There was a significant difference between the GnRH(+) and GnRH(-) groups including both sexes at week 6 (Fig 3A). In the females in Fig 3B, both GIs of the GnRH(+) and GnRH(-) groups gradually increased from week 2 to 6: A significant increase in the GnRH(-) group was observed from week 2 to 6, whereas no significant differences were seen between the GnRH(+) and GnRH(-) groups at any sampling time. In the males in Fig 3C, the GI of the GnRH(-) group slowly increased and peaked in week 4 at 16.6%, whereas the GI in the GnRH(+) group was elevated at week 4 with a peak (24.8%) in week 6 with a significant difference. Histological analysis revealed that females in the GnRH(+) group whose sex had been identified by gonad color were hermaphrodites. The GI of hermaphrodites treated with GnRH(+) was gradually increased from week 2 to 4 (Fig 3D).

### Changes in oocyte diameter and number of spermatogonia

**Fig 4:**
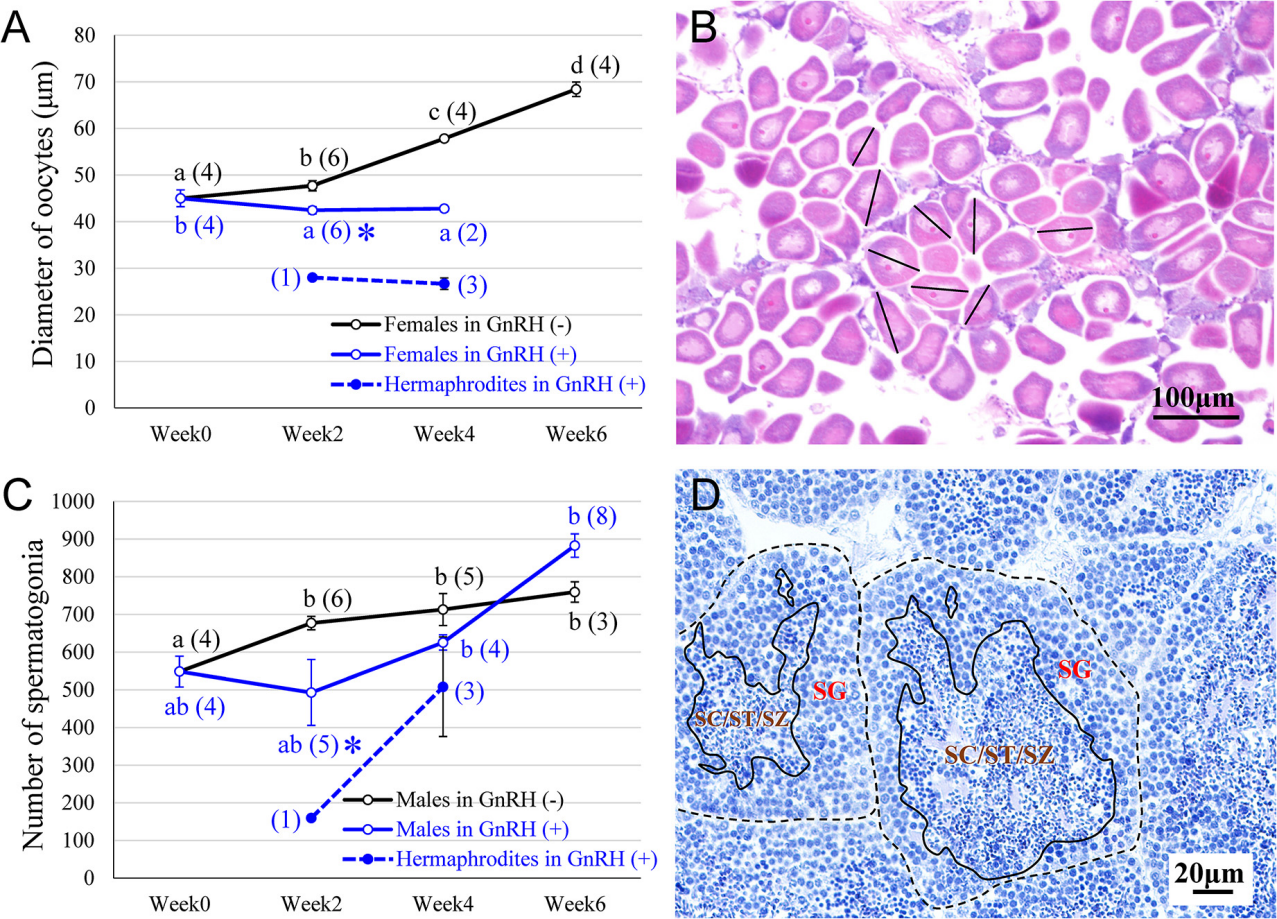
Changes in cell diameter of oocytes and cell number of spermatogonia in the gonad of scallops after py-GnRH administration during 6 weeks. A) Comparison of oocyte diameters of females or hermaphrodites between the GnRH(+) and GnRH(-) groups. B) The black lines show typical measurements of oocyte cell diameter in the long axis direction in the ovary. C) Shift of cell number of spermatogonia of male or hermaphrodite scallops between the GnRH(+) and GnRH(-) groups. D) A representative example for the counting of spermatogonia (SG) in the testicular germinal acini encircled with dashed lines: The areas encircled with lines where the differentiated testicular germ cells (i.e., SC; spermatocytes, ST; spermatids, SZ; spermatozoa) present were excluded from spermatogonia counting. Black, blue, and blue-dashed lines for the GnRH(-), GnRH(+), and hermaphrodites in the GnRH(+) groups, respectively. All data in A and C are presented as a mean ± standard error of the mean. Different superscript letters indicate significant differences within the same treatment group. Asterisks (*) indicate significant differences in the GnRH(+) group compared to GnRH(-) group at a particular sampling point at p < 0.05.

To assess the maturity of gonads, oocyte diameter was measured for evaluating ovarian development and the number of spermatogonia in each germinal acinus was counted for estimating testicular development. The oocyte diameter of females in the GnRH(-) group significantly increased from 45.0 to 68.4 μm during the 6 weeks, indicating that oocyte growth continued throughout the experiment. However, the oocytes of the GnRH(+) group had the same diameter (about 42–45 μm) during 4 weeks (Fig 4A). The hermaphrodites found in the GnRH(+) group possessed much smaller oocytes at around 26–28 μm diameter in weeks 2 and 4. Meanwhile, the average number of spermatogonial cells per germinal acinus of the GnRH(-) group increased from 549 to 760 cells in 6 weeks. While spermatogonial number did not change in the GnRH(+) group in the first 2 weeks, then it was drastically elevated to 883 cells by week 6. The hermaphrodite gonad found in the GnRH(+) group had fewer spermatogonia at around 160 cells in week 2, whereas there was an elevated number (510 cells) in week 4.

### Detection of apoptotic cells in the gonads of the GnRH(+) group

**Fig 5:**
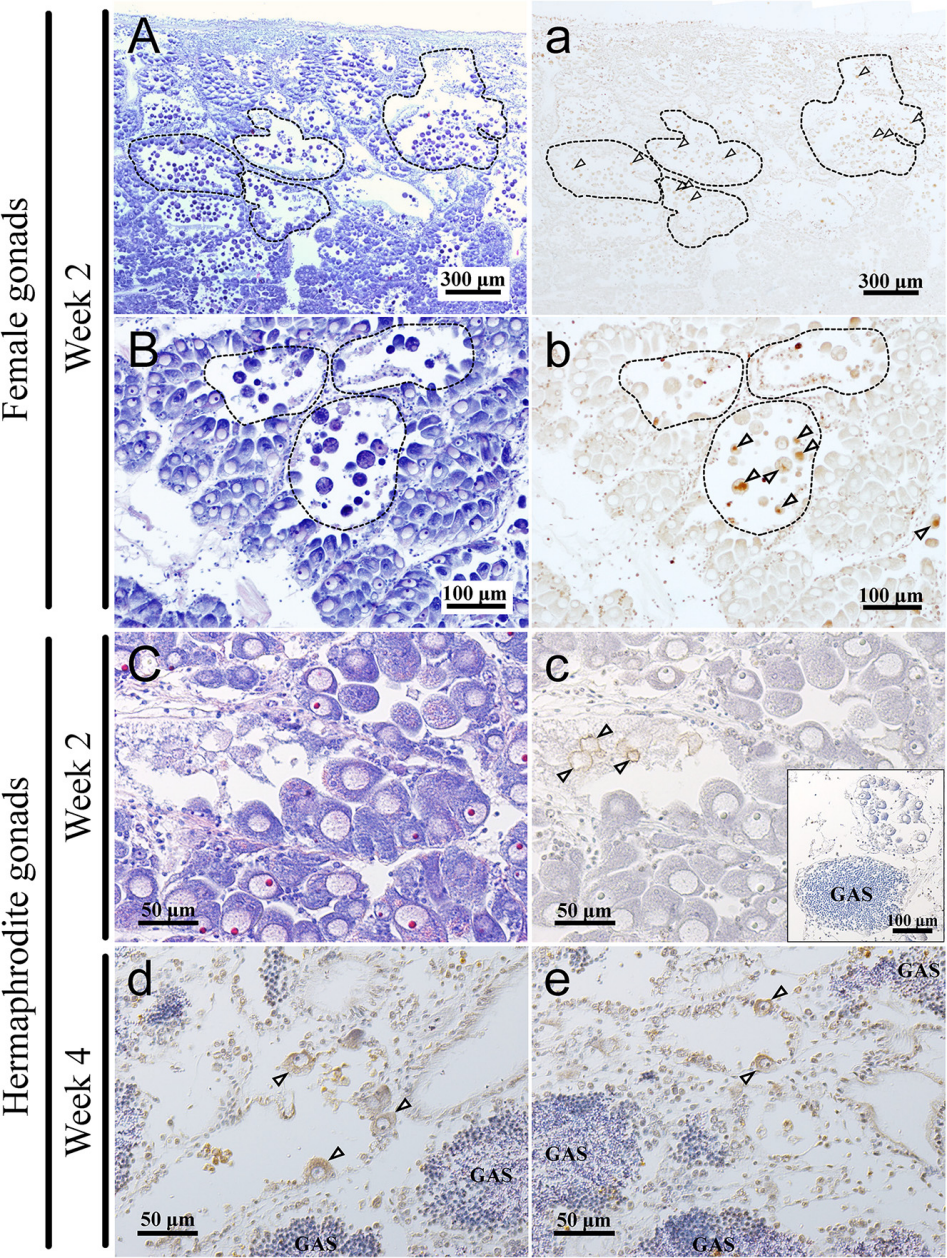
Detection of apoptotic cells in the gonads of the scallops after py-GnRH administration in week 2 and 4. The female gonads of GnRH(+) group in week 2 (A, a, B, b) and hermaphrodite gonads of the GnRH(+) group in week 2 (C, c) and 4 (d, e). A-C) Adjacent sections of a-c were stained by H.E. a-e) The sections were immunostained with DAB substrate to detect apoptotic cells. A, a, B, b) The germinal acini containing abnormal spaces in the ovary are encircled with dashed lines. a-e) Apoptotic cells found in the gonads of GnRH(+) are indicated with arrowheads. GAS; germinal acinus containing spermatogenic cells. The inset in c was captured from another area in the same section indicating that both GAS and germinal acinus containing oocytes exist.

To assess the gonad structure with germ cell morphology in the GnRH(+) group, histological analyses with H.E. staining and the TUNEL assay for detecting apoptotic cells were carried out. Among all gonads of the GnRH(+) group assessed, apoptotic cells were found in the female gonads in week 2 (Fig 5A and 5B) and the hermaphrodite gonads in week 2 and 4 (Fig 5C–5E). In the female gonads, the germinal acini containing oocytes with germinal vesicles were observed to have normal ovarian development, whereas some germinal acini had abnormal spaces caused by disappearance of oocytes from the germinal acinus (Fig 5A and 5B), despite the fact that they were mainly at the pre-spawning phase. Especially in the degenerating germinal acinus, most oocytes had a densely-stained cytoplasm, and the oocytes with a well-defined germinal vesicle were not found (Fig 5B). In addition, adjacent sections stained by TUNEL revealed that the densely-stained oocytes were mainly apoptotic cells (Fig 5B). In the hermaphrodite gonads possessing both oocytes and the germinal acini containing spermatogenic cells (Fig 5C inset, d, e), oocyte apoptosis was also observed in week 2 and 4 (Fig 5C–5E).

## Discussion

### In vivo administration of py-GnRH peptides into scallop gonads

The present study evaluated physiological effect of the undecapeptide form which is the most likely to be a truer form of the mature py-GnRH peptide in the scallop rather than dodecapeptide form [17] as a preliminary investigation. An additional experiment can consider precise difference among those two forms with their control peptides which consist of scrambled sequences of py-GnRH peptides. Though the studies reported the delivery method for dsRNA to scallop [20] and oyster [21], no administration method for a slow-release peptide delivery has been established in bivalves yet. Cacao butter is one of the most widely used substrates for the slow-release delivery system of peptides such as GnRH analogs (GnRHa) in teleosts [22, 23]. We hereby confirmed that its validity with scallops for 6 weeks. Notably, the injected scallops grew without any abnormality during the experiment, implying that this method can be a peptide delivery system for bivalves. Nevertheless, some mortality occurred after injection. This might be due to the injection position (Fig 1B) where the solidified substrate cut off the gonad intestinal loop [24], resulting in poor nutrient supply and decreased excretion. Hence, it would be better to avoid the injection into the gonad intestinal loop to reduce the mortality rate. In addition, the immunoquantification analysis by TR-FIA (S1 Fig) also suggested the possible presence of the released py-GnRH peptide detected from the hemocyte extracts. Hence we will further need to measure short-term release profile of py-GnRH administration with cacao butter method within a week after injection to analyze the acute change of py-GnRH level in hemolymph.

### Promotion of spermatogenesis accelerated by administration of py-GnRH

A major effect of py-GnRH administration was the promotion of spermatogenesis but not oogenesis. In *P*. *yessoensis*, we already revealed that the increase of spermatogonial cell number or oocyte diameter reflects testicular or ovarian development with changes in GI, respectively [25]. This accelerated increase of the GI in males by py-GnRH administration may be attributed to higher cell proliferation activity of spermatogonia and subsequent meiotic division resulting in more male gametes. In fact, our cell counting experiment revealed that a significant increase in the number of spermatogonia occurred in the GnRH(+) group over 6 weeks. This result is consistent with the in vitro studies that py-GnRH peptides could stimulate spermatogonial proliferation in the organ-cultured testis of scallops [5, 17]. This direct stimulatory effect of py-GnRH to spermatogonial proliferation may be a common effect among the bivalves since similar phenomenon were seen in *C*. *gigas* and *Mytilus edulis* as is the study with vertebrate-type GnRHs [6]. In addition, the expression analysis showing localization of an invGnRHR in *C*. *gigas* gonads [26] also implied physiological role of invGnRH related to bivalve reproduction. Meanwhile, a bioactive analysis based on the criteria of proliferative activity of py-GnRH inferred an alternative py-GnRH signaling pathway mediated with hemocytes as a carrier [5, 19]. Whatever the case, invGnRH present with a reproductive role in scallop as with octopus [12–15]. However, further molecular identification of scallop GnRHR homolog is surely needed to clarify the direct py-GnRH signaling pathway on spermatogenesis.

### Inhibitory role of py-GnRH on oogenesis

We also found that oocyte growth with healthiness in the GnRH(+) group could be affected by py-GnRH administration. TUNEL analysis revealed that apoptotic oocytes were partially found in the gonads of GnRH(+) group. Similar induced apoptosis caused by GnRH administration was previously reported in rat corpus luteal cells [27], human and porcine granulosa cells [28]. These direct local effect of a GnRH agonist resulting in apoptosis have been thought to be acting via GnRHR since these GnRHR mRNAs are localized in granulosa cells and luteal cells [29, 30]. To verify the apoptosis mechanism occurred in our specimens, further expression analysis of scallop GnRHR homolog in the gonads would be required. However, neither the GnRHR nor granulosa cells/steroid synthesizing cells have been characterized in scallops. Hence, a molecular marker gene-assisted cell characterization will also be thirsted for scallop gonad somatic cells for understanding the special cellular interactions between somatic and germ cells.

### Shifts of sex ratio of py-GnRH-administered scallops

Another physiological effect of py-GnRH administration in the present study was that the sex ratio seemed to be shifted to a male bias during the 6-week study, whereas the control group had an equal sex ratio throughout the experiment as is the case with the average of sex ratio in natural population of the northern coast of Japan [31]. It is noteworthy that some hermaphrodites (i.e., 8.3% in week 2 and 33.3% in week 4) were emerged in the GnRH(+) group. Generally, hermaphrodites have been rarely observed in the natural environment (not more than 0.3–0.4% of all cases [31]). In addition, Yesso scallop is protandrous [32] that has a mechanism to feminize from the male gonad by endocrinological regulation. Therefore it can be a reasonable assumption that our treatment could influence somewhat in the mechanism of sex reversal (i.e., masculinization) which is never seen in the natural environment. However, our additional investigations are surly needed to clarify the py-GnRH function on sex reversal. For instance, one can design another experimental setup with the scallops sorted by sexing prior to administration and subsequent chronological observation. If testis- or ovary-specific marker gene were identified in scallop, a transitional phase of masculinization could be detected by analyzing gene expression of those maker genes. Induction of sex reversal by steroid administration has been studied in *C*. *gigas* [33] and coot clam *Mulinia lateralis* [34]. More recent study reported that the injections of P, T, E_2_, or dehydroepiandrosterone (DHEA) all accelerated gonadal differentiation and influenced their sex ratio, with the males becoming more dominant in sea scallop *Placopecten magellanicus* [35]. Therefore, it can be expected that our specimens of the GnRH(+) group might be also in a condition where the production of some kind of sex steroids were accelerated by py-GnRH administration.

The other observation implying sex conversion in this study was that many apoptotic oocytes were detected in the hermaphrodites and a few of females in the GnRH(+) group, insinuating a degenerative process in loss of femininity and subsequent masculinization. A similar phenomenon has been reported in the other scallop species subjected to an injection of T, resulting in induced degeneration in oocytes of the females, whereas no effect was seen in the T-injected males, but hermaphrodites appeared in the P- or DHEA-injected groups [35]. These findings may support a hypothesis that some kind of sex steroids act on gonad development and may also be controlling sex differentiation in bivalves, even though there has been no convincing evidence whether vertebrate-type sex steroids exist in invertebrates [36, 37]. The recent genomic survey in mollusks have found the absence of the genes of key catalytic enzyme that conceptually acts in the pathway of vertebrate sex steroid biosynthesis (e.g., CYP19, an aromatase [38]) or the gene of a functional nuclear receptor that can bind with vertebrate-type sex steroids (i.e., a member in nuclear receptor 3 (NR3) group which can interact with vertebrate estrogen [39]). Nevertheless, vertebrate sex steroids showed certain effect on the mollusk physiology including reproductive function [40]. Due to these observations, the possible presence of vertebrate-like steroidogenesis in mollusk is still proposed to date [41, 42]. Altogether, it comes down to the mystery whether mollusks can transform the vertebrate steroids in good yield, and this can be analyzed by mass spectrometry suggested by Scott [37]. In terms of our experiment, immunoassayable steroids (e.g., P, E_2_, and T) can be quantified in the subjected scallops to see the endogenous steroid production influenced by py-GnRH administration, but it will arise the argumentation regarding the interference of non-specific assay for detecting mollusk steroids and the dubious steroidogenic pathway in mollusk mentioned above. Hence the present study leaves the discussion for the effect of sex steroids to be further investigated.

## Conclusion

InvGnRH and its receptor homologs have been cloned in a large variety of taxa including amphioxus, ascidians, an echinoderm, annelids, and mollusks (Roch et al., 2014a). This indicates that neuroendocrinological regulations by ancestral GnRH signaling is a widely-adopted system in most invertebrates. However its multifunctional role have been discursively reported in different mollusk species to have both non-reproductive and reproductive function. To be a part of knowledge for invGnRH function in mollusk, the present study performed py-GnRH administration with a slow-release delivery system that could provide preliminary evidence of invGnRH functions in scallop reproduction. This study helps us to understand the physiological role of invGnRH in scallop reproduction. Specifically, our study developed a hormonal administration method for scallops and revealed the in vivo effect of py-GnRH administration on scallop reproduction. This was an acceleration in spermatogenesis with a significant increase in the number of spermatogonia and GI in the males during the reproductive season. We also found an inhibitory effect of py-GnRH administration on oocyte growth in the females, implying early phenotypic alteration to masculinization. Thus, the method developed in this study can be utilized for a planned control of sexual maturity of male scallop bloodstock in advance of artificial seed production. Moreover, these findings will provide useful insights into invGnRH function in the molluskan endocrine system.

## Supporting Information

S1 Fig Immunoquantification of py-GnRH peptide in plasma and hemocyte extracts of the scallops in GnRH(+) and GnRH(-) groups in week 2 by time-resolved fluoresce immunoassay (TR-FIA). The TR-FIA procedure for the py-GnRH peptide quantification was preformed based on the established protocol as detailed elsewhere (Amano et al., 2011). In brief, the polyclonal antibody was raised against BSA-conjugated py-GnRH peptide: CQNFHYSNGWQP-NH_2_, and then the antiserum was affinity-purified with solid-phased py-GnRH peptide (Sigma-Aldrich, St. Louis, MO, USA) and used for TR-FIA validation. The sensitivity of the TR-FIA assay was validated with a ten-fold serial dilution (10^–6^ to 10^–11^ M) of the synthetic py-GnRH peptide as used in the peptide administration. For the sample measurement (GnRH(+): male n = 4; female n = 4, GnRH(-): male n = 4; female n = 4), withdrawn hemolymph was separated to plasma and hemocytes by centrifugation. Hemocytes were collected from whole hemolymph (approximately 2 to 3 ml) and homogenized with 500 μl of distilled water. Plasma (1 ml) or hemocyte extract (500 μl) was freeze-dried and reconstituted with the assay buffer (500 μl) and then individually subjected to measurement. Samples were measured in triplicate. In the S1 Fig, the standard carve ensures that this assay could detect the amount more than 10^–11^ M of py-GnRH peptide in the specimen. In hemolymph, py-GnRH was not detected in any plasma samples double strength of both groups, but a few of hemocyte extracts of the GnRH(+) group exhibited the concentration at 10^–9^ to 10^–10^ M (i.e., approximately 0.61 and 0.86 ng/ml). The data of samples in week 0, 4, and 6 are not shown due to no difference between the GnRH(+) and GnRH(-) groups.
(PDF)

S1 Table Chi-square test for the sex ratios of scallops in GnRH(-) and GnRH(+) groups during 6 weeks. (DOCX)
