# Supplementary figures and images for: In Vivo Administration of Scallop GnRH-Like Peptide Influences on Gonad Development in the Yesso Scallop, Patinopecten yessoensis
Source: PLoS One. 2015 Jun 1;10(6):e0129571. doi: 10.1371/journal.pone.0129571 (PMC4451010; doi:10.1371/journal.pone.0129571)

S1 Figure

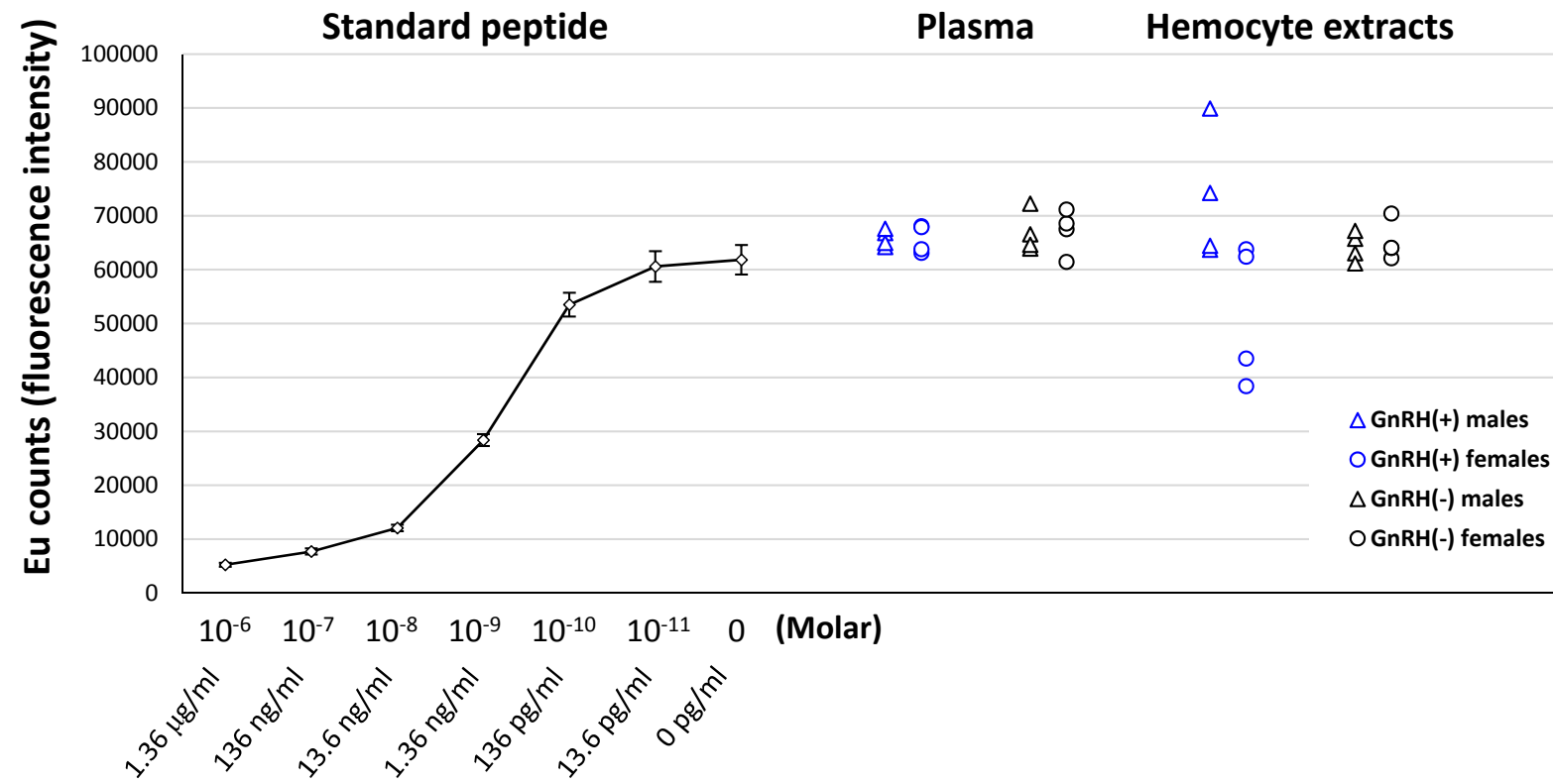

Supplement: S1 Fig — The TR-FIA procedure for the py-GnRH peptide quantification was preformed based on the established protocol as detailed elsewhere (Amano et al., 2011). In brief, the polyclonal antibody was raised against BSA-conjugated py-GnRH peptide: CQNFHYSNGWQP-NH2, and then the antiserum was affinity-purified with solid-phased py-GnRH peptide (Sigma-Aldrich, St. Louis, MO, USA) and used for TR-FIA validation. The sensitivity of the TR-FIA assay was validated with a ten-fold serial dilution (10–6 to 10–11 M) of the synthetic py-GnRH peptide as used in the peptide administration. For the sample measurement (GnRH(+): male n = 4; female n = 4, GnRH(-): male n = 4; female n = 4), withdrawn hemolymph was separated to plasma and hemocytes by centrifugation. Hemocytes were collected from whole hemolymph (approximately 2 to 3 ml) and homogenized with 500 μl of distilled water. Plasma (1 ml) or hemocyte extract (500 μl) was freeze-dried and reconstituted with the assay buffer (500 μl) and then individually subjected to measurement. Samples were measured in triplicate. In the S1 Fig, the standard carve ensures that this assay could detect the amount more than 10–11 M of py-GnRH peptide in the specimen. In hemolymph, py-GnRH was not detected in any plasma samples double strength of both groups, but a few of hemocyte extracts of the GnRH(+) group exhibited the concentration at 10–9 to 10–10 M (i.e., approximately 0.61 and 0.86 ng/ml). The data of samples in week 0, 4, and 6 are not shown due to no difference between the GnRH(+) and GnRH(-) groups. (PDF) [file pone.0129571.s001.pdf]
