# Supplementary material for: In Vivo Administration of Scallop GnRH-Like Peptide Influences on Gonad Development in the Yesso Scallop, Patinopecten yessoensis
Source: PLoS One. 2015 Jun 1;10(6):e0129571. doi: 10.1371/journal.pone.0129571 (PMC4451010; doi:10.1371/journal.pone.0129571)
Supplement: S1 Table — (DOCX) [file pone.0129571.s002.docx]

**Supporting information**

**S1 Table.** **Chi-square test for the proportion of sex ratio of scallops in GnRH(-) and GnRH(+) groups during six weeks.**

|  |  | **GnRH(-)** | | | | |  | **GnRH(+)** | | | | |
| --- | --- | --- | --- | --- | --- | --- | --- | --- | --- | --- | --- | --- |
|  |  | Week 0 | Week 2 | Week 4 | Week 6 | Total |  | Week 0 | Week 2 | Week 4 | Week 6 | Total |
| Females | Count | 4 | 6 | 4 | 4 | 18 |  | 4 | 6 | 2 | 0 | 12 |
|  | Percent of column total | 22.2 | 33.3 | 22.2 | 22.2 |  |  | 33.3 | 50.0 | 16.7 | 0 |  |
|  | Expected values | 4.0 | 6.0 | 4.5 | 3.5 |  |  | 2.6 | 3.9 | 2.9 | 2.6 |  |
|  | Standardized residuals | 0 | 0 | -0.333 | 0.378 |  |  | 0.74 | 0.90 | -0.45 | -1.36 |  |
| Males | Count | 4 | 6 | 5 | 3 | 18 |  | 4 | 5 | 4 | 8 | 21 |
|  | Percent of column total | 22.2 | 33.3 | 27.8 | 16.7 |  |  | 19.0 | 23.8 | 19.0 | 38.1 |  |
|  | Expected values | 4.0 | 6.0 | 4.5 | 3.5 |  |  | 4.5 | 6.8 | 5.1 | 4.5 |  |
|  | Standardized residuals | 0 | 0 | 0.333 | -0.378 |  |  | -0.442 | -1.209 | -0.854 | 2.828 |  |
| Hermaphrodites | Count | 0 | 0 | 0 | 0 | 0 |  | 0 | 1 | 3 | 0 | 4 |
|  | Percent of column total | 0 | 0 | 0 | 0 |  |  | 0 | 25.0 | 75.0 | 0 |  |
|  | Expected values | 0 | 0 | 0 | 0 |  |  | 0.9 | 1.3 | 1.0 | 0.9 |  |
|  | Standardized residuals | 0 | 0 | 0 | 0 |  |  | -0.343 | -0.096 | 0.758 | -0.343 |  |
| Total |  | 8 | 12 | 9 | 7 | 36 |  | 8 | 12 | 9 | 8 | 37 |
| Mortality |  | 0 | 8 | 10 | 2 | 20 |  | 0 | 11 | 10 | 1 | 22 |
|  |  |  |  |  |  |  |  |  |  |  |  |  |
|  |  | Chi-square = 0.254 with 3 df | | |  |  |  | Chi-square = 14.23 with 6 df | | |  |  |
|  |  | p = 0.96844 | |  |  |  |  | p = 0.027172 | |  |  |  |
